# Developmental patterns of DR6 in normal human hippocampus and in Down syndrome

**DOI:** 10.1186/1866-1955-5-10

**Published:** 2013-04-24

**Authors:** Anand Iyer, Jackelien van Scheppingen, Jasper Anink, Ivan Milenkovic, Gabor G Kovács, Eleonora Aronica

**Affiliations:** 1 Department of (Neuro)Pathology, Academic Medical Center, University of Amsterdam, Meibergdreef 9, Amsterdam, AZ 1105, The Netherlands; 2 Institute of Neurology, Medical University of Vienna, Vienna, Austria; 3 SEIN – Stichting Epilepsie Instellingen Nederland, Heemstede, The Netherlands; 4 Swammerdam Institute for Life Sciences, Center for Neuroscience, University of Amsterdam, Amsterdam, The Netherlands

**Keywords:** Alzheimer’s disease, APP, Death receptor 6, Development, Down syndrome, Hippocampus, Neurodegeneration

## Abstract

**Background:**

Death receptor 6 (DR6) is highly expressed in the human brain: it has been shown to induce axon pruning and neuron death via distinct caspases and to mediate axonal degeneration through binding to N-terminal β amyloid precursor protein (N-APP).

**Methods:**

We investigated the expression of DR6 during prenatal and postnatal development in human hippocampus and temporal cortex by immunocytochemistry and Western blot analysis (118 normal human brain specimens; 9 to 41 gestational weeks; 1 day to 7 months postnatally; 3 to 91 years). To investigate the role of N-APP/DR6/caspase 6 pathway in the development of hippocampal Alzheimer’s disease (AD)-associated pathology, we examined DR6 immunoreactivity (IR) in the developing hippocampus from patients with Down syndrome (DS; 48 brain specimens; 14 to 41 gestational weeks; 7 days to 8 months postnatally; 15 to 64 years) and in adults with DS and AD.

**Results:**

DR6 was highly expressed in human adult hippocampus and temporal cortex: we observed consistent similar temporal and spatial expression in both control and DS brain. Western blot analysis of total homogenates of temporal cortex and hippocampus showed developmental regulation of DR6. In the hippocampus, DR6 IR was first apparent in the stratum lacunosum-moleculare at 16 weeks of gestation, followed by stratum oriens, radiatum, pyramidale (CA1 to CA4) and molecular layer of the dentate gyrus between 21 and 23 gestational weeks, reaching a pattern similar to adult hippocampus around birth. Increased DR6 expression in dystrophic neurites was detected focally in a 15-year-old DS patient. Abnormal DR6 expression pattern, with increased expression within dystrophic neurites in and around amyloid plaques was observed in adult DS patients with widespread AD-associated neurodegeneration and was similar to the pattern observed in AD hippocampus. Double-labeling experiments demonstrated the colocalization, in dystrophic neurites, of DR6 with APP. We also observed colocalization with hyper-phosphorylated Tau and with caspase 6 (increased in hippocampus with AD pathology) in plaque-associated dystrophic neurites and within the white matter.

**Conclusions:**

These findings demonstrate a developmental regulation of DR6 in human hippocampus and suggest an abnormal activation of the N-APP/DR6/caspase 6 pathway, which can contribute to initiation or progression of hippocampal AD-associated pathology.

## Background

Death receptors (DRs) belong to the tumor necrosis factor receptor superfamily and are known to induce apoptosis (programmed cell death) via the intracellular portion of the receptor referred to as the ‘death domain.’ [1-3]. A growing body of evidence indicates that DRs may mediate a variety of biological functions, including nonapoptotic functions [1,4-7].

Tumor necrosis factor TNFRSF21 (death receptor 6, DR6) is a relatively new member of the DR family, which has been found to induce apoptosis when overexpressed [8,9] (for review see [3,7]). Recently, Nikolaev and colleagues [10] reported that the N-terminal fragment of amyloid precursor protein (N-APP) may act as a ligand of DR6 and trigger axon pruning and neurodegeneration via caspase 6, suggesting a role for this receptor in the neurodegeneration observed in Alzheimer’s disease (AD; for review see [11]). Interestingly, different signaling cascades have been shown to be involved in degeneration of the axon (caspase 6) and neuronal cell body (caspase 3) [10,12]. Although there is still discussion about the formation of the N-APP fragment [13,14] and the structural features of the potential DR6-APP signaling complex [15,16], the identification of this APP-dependent pathway involving DR6 highlights the potential role of this receptor in specific types of disease-associated axonal degeneration. In addition, recent studies suggest additional physiological functions of DR6 during brain development [5,6].

DR6 is expressed in most human tissues [7,8]. Interestingly, DR6 mRNA expression is high in adult brain and particularly enriched in regions like the hippocampus that are vulnerable in AD [10]. The downstream DR6 effector, caspase 6, has been shown to be activated early in AD and to be associated with mild cognitive impairment [17-19]. However, the expression pattern and cellular localization of DR6 protein during human brain development remains uncharacterized.

In this study, we examined the expression of DR6 in the developing human hippocampus and temporal cortex to get a better insight into the role of this receptor in prenatal human brain development. In addition, we investigated DR6 expression in the developing hippocampus of patients with Down syndrome (DS) prior to establishment of AD neurodegeneration and in DS patients with AD pathology compared with age-matched control, and in hippocampal specimens obtained from patients with sporadic AD with severe, endstage pathology. Elucidation of complex pathogenetic pathways characterizing the earliest stage of the detrimental processes that result in neurodegeneration represents an essential first step towards a therapeutic intervention, which could be able to block these pathological processes and, eventually, prevent the onset of the disease in DS patients.

## Methods

### Human material

The subjects included in this study were selected from the databases of the Departments of Neuropathology of the Academic Medical Center, University of Amsterdam (in a project entitled ‘Pathways common to brain development and aging: defining strategies for preventive therapy and diagnostics’ approved by the Research Ethics Committee of the Academic Medical Center), and the Institute of Neurology, Medical University of Vienna, Austria (in a project approved by the Ethical Committee of the Medical University of Vienna, entitled ‘Molecular neuropathologic examinations of neurodegeneration-related proteins in Down syndrome, Ek Nr. 1316/2012), from the NICHD Brain and Tissue Bank for Developmental Disorders and from the Netherlands Brain Bank. Informed consent was obtained for the use of brain tissue and for access to medical records for research purposes. Tissue was obtained and used in a manner compliant with the Declaration of Helsinki. We included brains of fetuses at different gestational ages (9 to 36 gestational weeks (GW)), neonates, and children from control and DS patients (Table 1). Fetal brain was obtained from spontaneous or medically induced abortions with appropriate maternal written consent for brain autopsy. Gestational ages were based on obstetric data, fetal and brain weights and standard fetal anthropometric measurements. We performed a careful histological and immunohistochemical analysis and evaluation of clinical data (including genetic data, when available). We excluded subjects with other chromosomopathies, major central nervous system malformations, brains with postmortem autolysis, severe hypoxic or ischemic encephalopathy, intraventricular hemorrhages, severe hydrocephalus, and meningitis or ventriculitis. Dyslamination in the dentate gyrus was observed in one DS subject. As controls, we only included brain specimens displaying a normal hippocampal and cortical structure for the corresponding age and without any significant brain pathology. We did not use quantitative methods in this study to evaluate gliosis or neuronal cells loss. We acknowledge the limitations of visual inspection alone, which does not accurately detect neuronal loss below approximately 30% cell loss.

**Table 1:** Cases included in this study

**Age range**	**Number of cases**	**Sex (male/female)**
**Controls**		
9 to 20 GW	36	19/17
21 to 41 GW	43	24/19
1 to 6 days	6	2/4
2 weeks to 7 months	21	11/10
3 to 65 years	18; 3 stage II	7/11
**Trauma**		
30 to 67 years	3	3/0
**Down syndrome**		
14 to 20 GW	20	9/6 5 not determined
21 to 41 GW	13	7/6
7 to 12 days	3	3
2 to 8 months	5	4/1
15 to 64 years	7; 3 stage V; 2 VI	male
**Alzheimer’s disease**		
77 to 90 years	6; 3 stage V; 3 VI	2/4

GW: gestational weeks; Braak Neurofibrillary Staging: Stage II-VI.

Additionally, we obtained adult brain tissue at autopsy from eleven control subjects (without evidence of degenerative changes, and lacking a clinical history of cognitive impairment), three patients with posttraumatic brain injury, six patients with DS (Braak Neurofibrillary Staging: V and VI), and nine patients with AD (three with Braak stage II, without signs of cognitive impairment and six with Braak stage V and VI) (Table 1). All subjects were pathologically staged according to Braak and Braak criteria [20]. Subjects without known cause of death were excluded. All autopsies were performed within 24 h after death.

### Tissue preparation

One or two representative paraffin blocks per brain (hippocampus and temporal cortex) were sectioned, stained, and assessed. Formalin-fixed, paraffin-embedded tissue was sectioned at 6 μm and mounted on precoated glass slides (Star Frost, Waldemar Knittel GmbH, Braunschweig, Germany). Sections of all specimens were processed for hematoxylin eosin, Luxol fast blue, and Nissl stains, as well as for immunocytochemical stains for a number of markers, listed next. We performed a silver impregnation (Bielschowsky) staining in all adult brains.

### Immunocytochemistry

Glial fibrillary acidic protein (GFAP; polyclonal rabbit, DAKO, Glostrup, Denmark; 1:4,000; monoclonal mouse; DAKO; 1:50), vimentin (mouse clone V9, DAKO; 1:400), neuronal nuclear protein (NeuN; mouse clone MAB377, IgG1; Chemicon, Temecula, CA, USA; 1:1,000), neurofilament protein (mouse clone 2 F11, Neomarkers, Fremont, CA, USA; 1: 200), synaptophysin (mouse clone Sy38; DAKO; 1:200; polyclonal rabbit, DAKO; 1:200), human leukocyte antigen(HLA)-DP, DQ, DR (major histocompatibility complex class II, MHC-II; mouse clone CR3/43; DAKO, Glostrup, Denmark, 1:400), and CD68 (mouse clone PG-M1, DAKO, Glostrup, Denmark; 1:200) were used in the routine immunocytochemical analysis.

To detect death receptor 6 (DR6), we used a polyclonal rabbit antibody (Santa Cruz Biotechnology, Santa Cruz, CA, USA; 1:100); to detect APP, a monoclonal antibody (mouse clone 22C11, Chemicon, Temecula, CA, U.S.A;1:50,000); and to detect caspase 6, a polyclonal rabbit antibody (Abcam, Cambridge, MA, USA;1:500).

Single-label immunocytochemistry was developed using the Powervision kit (Immunologic, Duiven, The Netherlands). 3,3-Diaminobenzidine (Sigma, St. Louis, USA) was used as chromogen. Sections were counterstained with hematoxylin.

For double labeling of DR6 with amyloid-β (clone M0872, DAKO; 1:200), GFAP, or synaptophysin, sections were, after incubation with the primary antibodies overnight at 4°C, incubated for 2 h at room temperature with Alexa Fluor® 568-conjugated anti-rabbit and Alexa Fluor®-conjugated 488 anti-mouse IgG or anti-goat IgG (1:100, Molecular Probes, The Netherlands). Sections were then analyzed using a laser scanning confocal microscope (Leica TCS Sp2, Wetzlar, Germany).

For double labeling of DR6 with APP or Tau (Clone AT8, Innogenetics Alpharetta, GA, USA; 1:5,000), or caspase 6 or caspase 3 (polyclonal rabbit, Signaling Technology Danvers, MA, USA; 1:100), sections were incubated with Brightvision poly-alkaline phosphatase-anti-rabbit (Immunologic, Duiven, The Netherlands) for 30 min at room temperature, and washed with PBS. Sections were washed with Tris–HCl buffer (0.1 M, pH 8.2) to adjust the pH. Alkaline phosphatase activity was visualized with the alkaline phosphatase substrate kit I Vector Red (SK-5100, Vector laboratories Inc., CA, USA). To remove the first primary antibody (DR6), sections were incubated at 121°C in citrate buffer (10 mM NaCi, pH 6.0) for 10 min. Incubation with the second primary antibody was performed overnight at 4°C. Sections with primary antibody other than rabbit were incubated with post antibody blocking from the Brightvision+ system (containing rabbit-anti-mouse IgG; Immunologic, Duiven, The Netherlands). Alkaline phosphatase activity was visualized with the alkaline phosphatase substrate kit III Vector Blue (SK-5300, Vector laboratories Inc., CA, USA). Sections incubated without the primary antibodies or with the primary antibodies, followed by heating treatment were essentially blank. Images were analyzed with a Nuance VIS-FL Multi-spectral Imaging System (Cambridge Research Instrumentation; Woburn, MA), as described previously [21,22].

### Evaluation of immunostaining

All labeled tissue sections were evaluated by two independent observers, blind to clinical data, for the presence or absence of various histopathological parameters and specific immunoreactivity for the different markers. The intensity of DR6 staining was evaluated, as previously described [23,24], using a using a semi-quantitative scale ranging from 0 to 4 (0: negative; 1: weak; 2: moderate; 3: strong; 4: very strong staining). Different sub-areas of the hippocampus (CA1 to CA4 and dentate gyrus) were examined: the score represents the predominant intensity found in each case.

We measured optical density in control and DS hippocampus (as previously described [25]) for DR6 in the CA1. Sections were digitized using an Olympus microscope equipped with a DP-10 digital camera (Olympus, Japan). Images from consecutive, nonoverlapping, fields (magnification, 20×) were collected using image acquisition and analysis software (Phase 3 Image System integrated with Image Pro Plus; Media Cybernetics, Silver Spring, MD). The absolute pixel staining density and the background from fields lacking labeling was determined. A mean optical density value for the CA1 was calculated, expressed as a ratio with the mean optical density of the background and comparison was made between patients. Statistical analyses were performed with SPSS for Windows (SPSS 11.5, SPSS Inc., Chicago, IL, USA). Data were analyzed using a two-tailed Student’s *t* test or a nonparametric Kruskal-Wallis test, followed by a Mann–Whitney test to assess the difference between groups. A value of *P* < 0.05 was defined statistically significant.

### Western blot analysis

For immunoblot analysis we used frozen brain specimens (control cortex: 13 to 41 GW; 1 day postnatally; 2 and 7 months postnatally; 3, 4, and 7 years; and adult cortex (46, 50, 50 years); as well as control hippocampus: 19 GW; 2 months postnatally; 2 years; and adult hippocampus). The frozen specimens were homogenized in lysis buffer containing 10 mM Tris (pH 8.0), 150 mM NaCl, 10% glycerol, 1% NP-40, 0.4 mg/ml Na orthovanadate, 5 mM EDTA (pH 8.0), 5 mM NaF, and protease inhibitors (cocktail tablets, Roche Diagnostics, Mannheim, Germany). Protein content was determined using the bicinchoninic acid method [26]. For electrophoresis, equal amounts of protein (50 μg/lane) were separated by sodium dodecylsulfate-polyacrylamide gel electrophoresis (SDS-PAGE) (10% acrylamide). Separated proteins were transferred to nitrocellulose paper by electroblotting for 1 h and 30 min (BioRad, Transblot SD, Hercules, CA). After blocking for 1 h in Tris-buffered saline with Tween (TBST; 20 mM Tris, 150 mM NaCl, 1% Tween, pH 7.5)/5% nonfat dry milk, blots were incubated overnight at 4°C with rabbit anti-DR6 (1:1,000), mouse anti-β-tubulin (1:30,000, monoclonal mouse, Sigma, St. Louis, MO, USA), or APP (1:50,000). After several washes in TBST, the membranes were incubated in TBST and 5% nonfat dry milk, containing the goat anti-rabbit or rabbit anti-mouse coupled to horse radish peroxidase (1:2,500; Dako, Denmark) for 1 h. After washing in TBST, immunoreactivity was visualized using ECL PLUS Western blotting detection reagent (GE Healthcare Europe, Diegen, Belgium). To quantify the blots, band intensities were measured densitometrically using Scion Image for Windows (beta 4.02) image-analysis software. A ratio of the integrated band density (IntDen) of the protein of interest to the IntDen of the reference protein was used to normalize band intensities.

## Results

### Developmental expression of DR6 in control hippocampus and cerebral cortex

**Figure 1:**
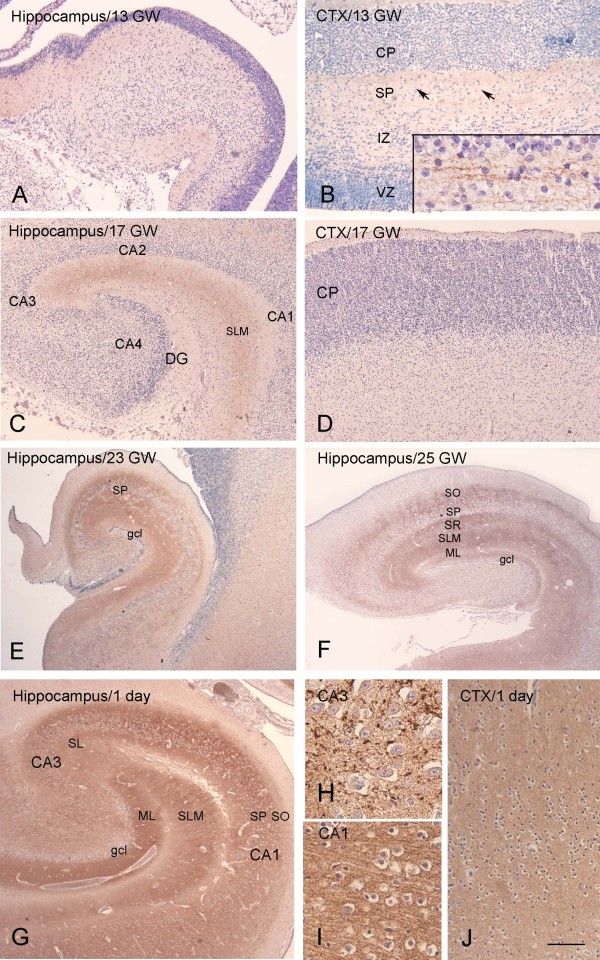
**DR6 immunoreactivity (IR) at different gestational ages (13, 17, 23, 25 gestational weeks, GW) and at 1 day postnatally. A-B**: 13 GW. **A:** the hippocampus shows no detectable DR6 IR. **B:** the cerebral cortex shows weakly reactive fibers in the subplate zone (SP, arrows; high magnification in insert), but no reactivity in the cortical plate (CP), intermediate zone (IZ) or ventricular zone (VZ). **C-D:** 17 GW. **C**: light DR6 IR is detected in the hippocampus in the stratum lacunosum-moleculare (SLM). **D:** cerebral cortex shows no reactivity in the CP. **E** (23 GW) and **F** (25 GW) show evident DR6 IR in the different hippocampal subfields (stratum oriens, SO; stratum pyramidale, SP; stratum lacunosum-moleculare, SLM; molecular layer dentate gyrus, ML). **G-J:** 1 day. **G:** strong DR6 IR is observed throughout the different hippocampal subfields; high magnification of CA3 and CA1 is shown in H and I, respectively. **J:** Diffuse and strong DR6 IR is observed throughout the neocortex. Dentate gyrus, DG; granule cell layer, gcl; stratum lucidum, SL. Hematoxylin counterstain shows blue nuclei. Scale bar (shown in J): A, C-G, 400 μm; B, 200 μm; H-I, 40 μm; J, 80 μm.

**Figure 2:**
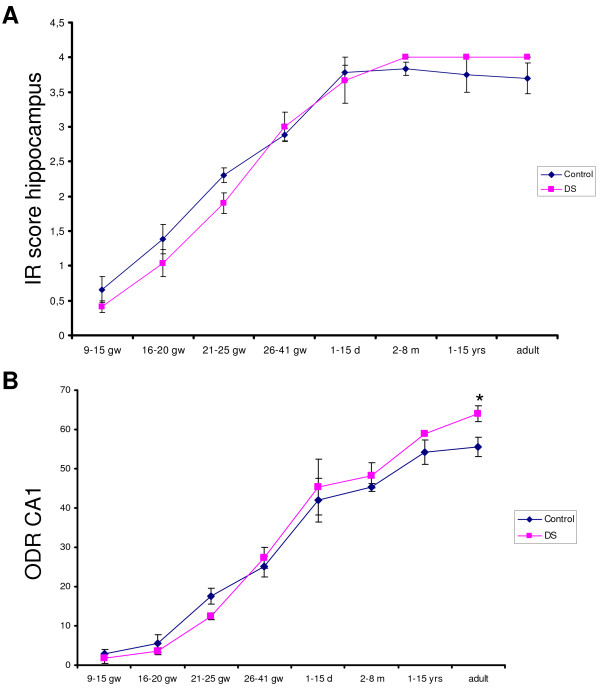
**Evaluation of DR6 immunoreactivity in hippocampus of control and Down syndrome (DS) during development. A:** immunoreactive score (IR) for DR6 in the hippocampus (CA1 to CA4 and dentate gyrus) **B:** relative optical density ratio (ODR) of DR6 immunoreactivity in CA1. Values represent the mean ± SEM of samples at different prenatal and postnatal ages. *P < 0.05, compared with control.

The expression pattern of DR6 was studied immunocytochemically at different prenatal ages, ranging from 10 to 40 GW, as well as at postnatal ages (1 day to 7 months and 3 to 8 years; Figure 1; Additional file 1: Figure S2). At the earliest stages of development evaluated (9 to 15 GW), DR6 expression in the hippocampus and cortical plate was below the level of detection (Figure 1A-B; Table 2). Weakly reactive fibers were, however, detected in the subplate at 13 to 15 GW (Figure 1D). In the hippocampus, DR6 IR was first apparent in the stratum lacunosum-moleculare at 16 GW, followed by the stratum oriens, radiatum, pyramidale (CA1 to CA4) and the molecular layer of the dentate gyrus between 21 and 25 GW (Figure 1C-F; Table 2). During this developmental period, an increase in DR6 IR was also detected in the neocortex, reaching a pattern similar to adult hippocampus around birth (Figure 1I). From 40 weeks to term, the entire hippocampal complex displayed prominent IR, with a pattern similar to that observed in adult hippocampus (Figure 1G-H; Additional file 2: Figure S1; Figure 2A). The IR pattern was similar in young adults (<40 years) compared with older control subjects (>50 years).

**Table 2:** Summary of DR6 immunoreactivity in human fetal hippocampus in control and Down syndrome

**Hippocampal complex**	**Onset staining**	**Onset staining**
	**Control (GW)**	**Down syndrome (GW)**
Stratum lacunosum-moleculare	16	16
Stratum oriens	21	21
Stratum radiatum	22	22
Stratum pyramidale (CA1 to CA4)	23	23
Molecular layer–dentate gyrus	23	23
Subiculum	23	23
Stratum lucidum	40	41

GW: gestational week.

**Figure 3:**
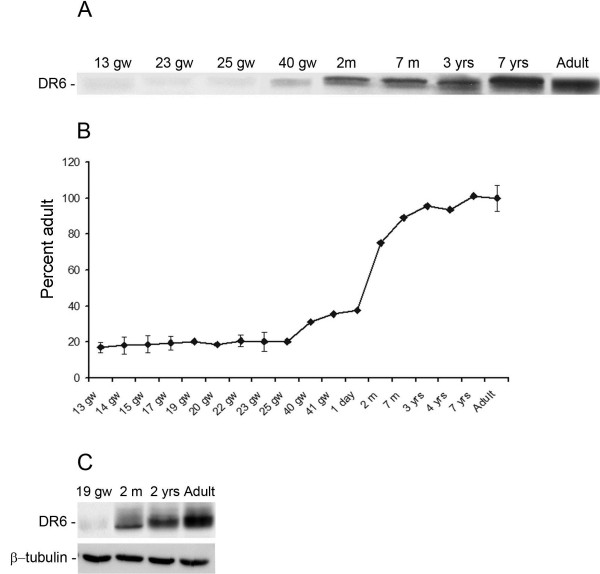
**Western blot analysis of DR6 in total homogenates of temporal cortex at different gestational ages. A:** representative blot of DR6 (≈75 kDa) in total homogenates of 13, 23, 25, 40 gestational weeks (GW) cortex, 2, 7 months, and 7 years postnatally and in adult temporal cortex. **B:** densitometric analysis. For each sample, the optical density of the DR6 was calculated relative to the optical density of the reference protein β-tubulin and expressed as percentage of adult control (mean ± SEM of three samples from 13, 14, 15, 17, 22, 23, 25 GW cortex, and control adult cortex). **C:** representative blot of DR6 and β-tubulin in total homogenates of 19 GW, 2 months, 2 years, and adult hippocampus.

Western blot analysis could only be performed where frozen temporal cortex and hippocampus material was available (Figure 3). As previously reported [27], DR6 receptor protein was detected as a band of approximately 75 kDa, consistent with the predicted size from DR6 cDNA. Expression of DR6 in the temporal cortex (Figure 3) and hippocampus (not shown) was detected at all ages examined; however, its expression increased postnatally in the cortex, reaching adult levels by about 1 year of age.

### Developmental expression of DR6 in Down syndrome hippocampus and cerebral cortex

**Figure 4:**
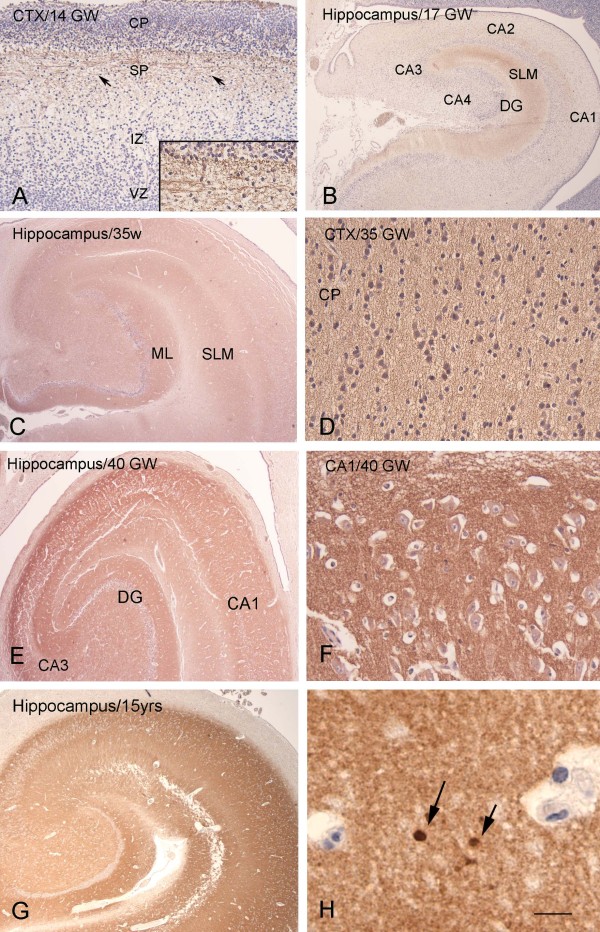
**DR6 immunoreactivity (IR) at different gestational ages (14, 17, 35, 40 gestational weeks, GW) in Down syndrome. A** 14 GW: the cerebral cortex shows weakly reactive fibers in the subplate zone (SP, arrows; high magnification in insert), but no reactivity in the cortical plate (CP), intermediate zone (IZ), or ventricular zone (VZ). **B** 17 GW: light DR6 IR is detected in the hippocampus in the stratum lacunosum-moleculare (SLM; DG, dentate gyrus). **C**-**D** (35 GW): DR6 IR is observed throughout the different hippocampal subfields (C) and in the neocortex (D). **E**-**F** 40 GW: strong DR6 IR is observed in the different hippocampal subfields (high magnification of CA1 is shown in F). **G**-**H** 15 years: with DR6 IR throughout the different hippocampal subfields and focal accumulation of IR in dystrophic neurites in the CA1 (arrows in **H**). Hematoxylin counterstain shows blue nuclei. Scale bar (shown in H): A, 200 μm; B, C, E, G, 400 μm; D, F, 40 μm; H, 20 μm.

In DS brains, the DR6 pattern of IR in both fetal hippocampus and neocortex was similar to that observed in age-matched controls (Figure 2A-B; Figure 4; Table 2). Only weakly reactive fibers were detected in the cortical subplate at 13 to 15 GW (Figure 4A). Between 21 and 35 GW the DR6 IR increased progressively in the neocortex (Figure 4D; 35 GW), reaching a pattern similar to that observed in adult hippocampus around birth (not shown). In the hippocampus, DR6 IR was first apparent in the SLM at 16 GW, followed by the stratum oriens, radiatum, pyramidale (CA1 to CA4), and molecular layer of the dentate gyrus between 21 and 35 GW (Figure 4B-C; Table 2). From 40 weeks to term, prominent IR was observed throughout the different hippocampal subfields with a pattern similar to that detected at postnatal ages (Figure 4E-G; Figure 2A). Increased DR6 expression was occasionally detected in dystrophic neurites in a 15-year-old DS patient (Figure 4H). The immunoreactivity score (in the hippocampus) and the relative ODRs (in the CA1) of fetal and infant DS samples were similar to age-matched controls (Figure 2A-B). Colocalization was observed with synaptophysin; we did not observe colocalization with GFAP (data not shown).

### Expression pattern of DR6 in DS with AD pathology

**Figure 5:**
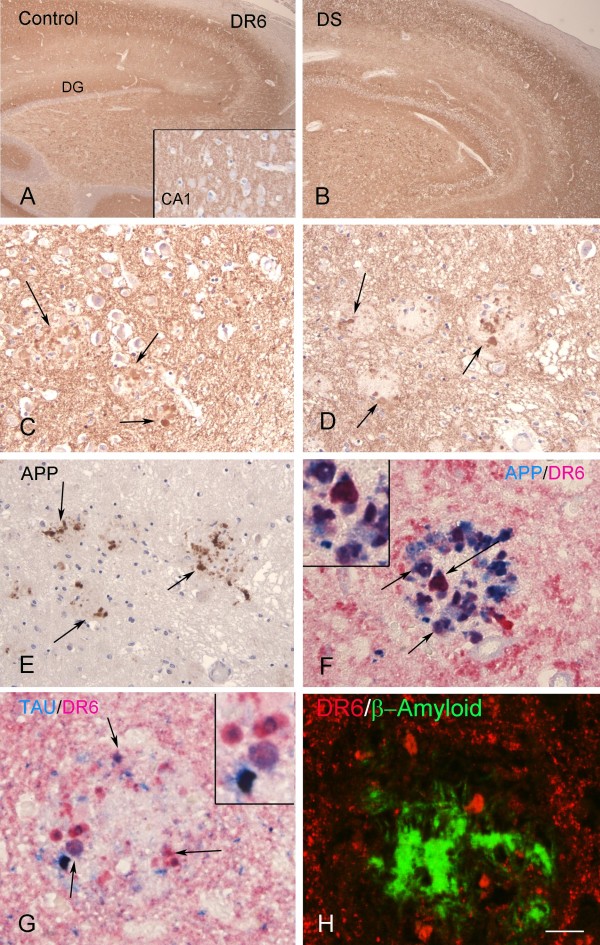
**DR6 immunoreactivity (IR) in control and Down syndrome (DS) adult hippocampus. A-B:** adult hippocampus of control (**A**) and DS (**B**), showing DR6 IR throughout the different hippocampal subfields (DG, dentate gyrus). **C, D:** high magnification of CA1 (C) and DG (D) in DS showing strong DR6 expression in dystrophic neurites in amyloid plaques (arrows). **E:** amyloid precursor protein (APP) IR is detected in the dystrophic neurites of plaques (arrows) in DS. **F:** colocalization of APP and DR6 in dystrophic neurites (purple; arrows) in DS. **G:** localization of hyperphosphorylated Tau and DR6 in dystrophic neurites (purple; arrows and insert) in DS. **H:** Confocal image showing accumulation of DR6 IR (red) around β-amyloid deposits (green) in DS. Hematoxylin counterstain shows blue nuclei (A-E). Scale bar (shown in H): A-B, 400 μm; C-E, 80 μm; F-H, 40 μm.

In adult hippocampus of 50 to 64-year-old DS, the presence of AD pathology was associated with an abnormal pattern of DR6 IR (Figure 5). Strong DR6 IR was detected in dystrophic neurites in and around amyloid plaques (Figure 5C-D). Although the immunoreactive score of adult hippocampus was similar to controls (Figure 2A), the ODR in the CA1 was higher in DS with AD pathology than in age-matched controls (Figure 2B). Evaluation of APP IR during hippocampal development showed increased IR in CA1 neurons prior to establishment of AD pathology (Additional file 1: Figure S2A-B). By Western blot analysis, a major band corresponding to the mature ~130 kDa isoform was identified at all ages examined (not shown). In DS brains with AD pathology, prominent increase of APP IR was detected throughout the hippocampus (Additional file 1: Figure S2C; Figure 5E). DR6 IR colocalized with APP and hyperphosphorylated Tau in plaque-associated dystrophic neurites but not with the β-amyloid core (Figure 5F-H).

Occasionally, DR6 IR was detected in dystrophic neurites in elderly patients (Braak stage II), without cognitive decline (not shown). The pattern of DR6 IR in the hippocampus of AD patients (Braak stage V-VI) was similar to that observed in DS with AD pathology (Additional file 3: Figure S3), with strong DR6 IR in dystrophic neurites in and around amyloid plaques (Additional file 3: Figure S3 B-D) and colocalization with APP and hyperphosphorylated Tau (Additional file 3: Figure S3 E-H). Spectral analysis of double labeling of DR6 with hyperphosphorylated Tau or with APP confirmed the colocalization in both AD and DS adult hippocampus (Additional file 4: Figure S4).

### Expression pattern of caspase 6 in DS with AD pathology: colocalization with DR6

**Figure 6:**
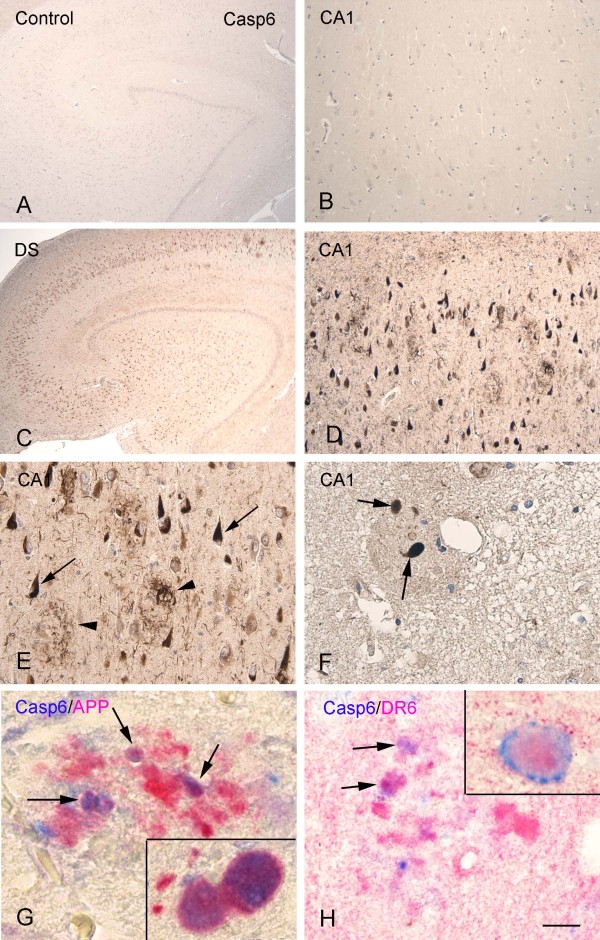
**Caspase 6 (Casp6) immunoreactivity (IR) in controls and Down syndrome (DS) adult hippocampus. A-B:** control hippocampus (B, CA1) without detectable Casp6 IR, without accumulation in neurons. **C-F:** DS hippocampus showing increased Casp6 IR throughout the hippocampus; D-F show high magnification of CA1 with strong IR in tangles (arrows in E), neuropil threads, and dystrophic neurites in plaques (arrowheads in E and arrows in F). **G:** colocalization of Casp6 and APP in dystrophic neurites (purple; arrows and insert). **H:** colocalization of Casp6 and DR6 in dystrophic neurites (purple; arrows and insert). Hematoxylin counterstain shows blue nuclei (A-F). Scale bar (shown in H): A,C, 400 μm; B, D, 160 μm; E, 80 μm; F, 40 μm. G-H, 25 μm.

**Figure 7:**
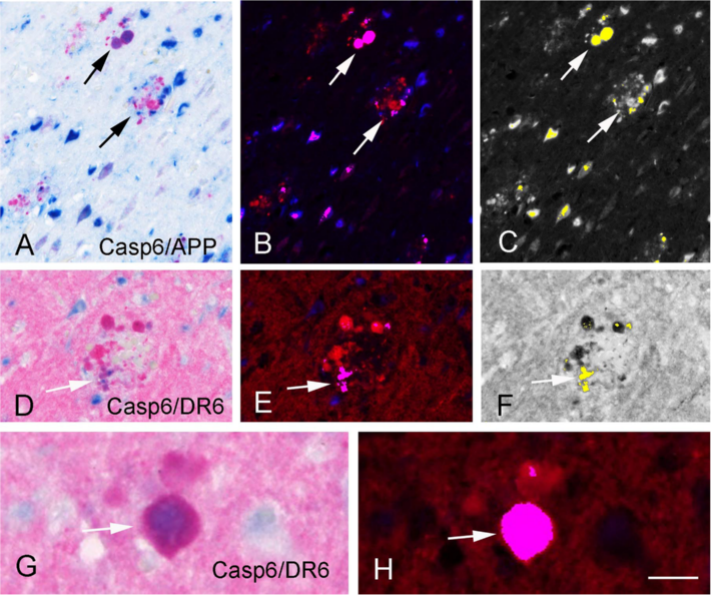
**Spectral analysis of double labeling of caspase 6 (Casp6) with Amyloid precursor protein (APP) or DR6 in Down syndrome (DS) adult hippocampus. A-C:** double labeling of Casp6 with APP. A: light microscopical image of the original immunostained section showing Casp6 in blue and APP in red. B, C: spectral analysis of the same section showing double stained structures in purple (arrows in B) and in yellow (arrows in C). **D**-**H:** double labeling of Casp6 with DR6. D, G: light microscopical image of the original immunostained section showing Casp6 in blue and DR6 in red. E, F, H: spectral analysis showing double stained structures in purple (arrows in E and H) and in yellow (arrow in F). Scale bar (shown in H): A-C, 80 μm; D-F, 40 μm; G-H, 20 μm.

In adult hippocampus of 50 to 64-year-old DS with AD pathology we observed prominent upregulation of caspase 6 IR throughout the hippocampus with strong expression in neuritic plaques and neurofibrillary tangles (Figure 6C-F). Caspase 6 IR colocalized with DR6 and APP in plaque-associated dystrophic neurites (Figure 6G-H); we did not observe colocalization of DR6 with caspase 3. Spectral analysis of double labeling of caspase 6 with APP and DR6 confirm colocalization in DS adult hippocampus with AD pathology (Figure 7), and in AD hippocampus (not shown).

## Discussion

Increasing evidence supports the concept that neurological disorders of the adult brain could represent a disorder of aberrant neural development. According to this ‘developmental hypothesis,’ even neurodegenerative disorders might have ‘fetal’ origins (for reviews see [11,28,29]).

Recent studies have provided evidence of a physiological function for N-APP in developmental pruning, which, together with other mechanisms, could also contribute to neurodegeneration in AD (for review see [11]). In particular, it has been suggested that activation of DR6 is required for initiation or progression of axonal degeneration [10]. However, the expression pattern and cellular localization of DR6 protein in developing brain is still largely uncharacterized and it is not clear whether, or to what extent, the N-APP-DR6 pathway might be abnormally activated in AD.

This study provides the first description of the expression pattern and cellular localization of DR6 in human hippocampus and neocortex during the pre- and early postnatal development. The period of prenatal development studied (9 to 40 GW) includes all the critical stages of human hippocampal and cortical development [30-32].

Developmental regulation of DR6 expression was observed in both neocortex and hippocampus by immunocytochemistry and was confirmed by Western blot analysis, revealing relatively high expression levels in adult brain tissue. In agreement with previous observations at the mRNA level [10], DR6 was particularly enriched in the hippocampus. DR6 IR was already detectable in the SLM at 16 GW and prominent IR was observed throughout the different hippocampal subfields by 40 GW. The expression of DR6 during the prenatal stages of hippocampal development supports the suggested physiological role of this receptor in shaping neuronal connections, in particular contributing to axonal pruning (for review see [11]), which deserves further investigation in experimental models.

An attractive hypothesis, provided by *in vitro* and *in vivo* studies using DR6 knockout mice, is that activation of DR6 by N-APP may, via caspase 6, contribute to degenerative processes [10]. To evaluate whether the N-APP/DR6/caspase 6 pathway is abnormally activated in AD and whether its activation might precede the development of AD, we studied the components of this pathway in DS hippocampus. AD-associated neuropathology is a consistent feature in DS patients and DS represents an opportunity to understand the link between early aging and neurodegenerative processes [33,34]. Immunocytochemical evaluation of APP (using an antibody that recognizes N-APP) provided evidence of expression prior to establishment of AD pathology. In addition, an increased expression of DR6 in dystrophic neurites (similarly to cases of early AD pathology; Braak stage II) was detected in a 15-year-old DS patient. Detection of a premature neuritic pathology in DS is consistent with previous evidence supporting the occurrence of ongoing neurodegeneration prior to the establishment of widespread AD neurodegeneration [35-38]. In adult DS cases with frank dementia as well as significant AD pathology, caspase 6 expression was strongly increased within the hippocampus. Interestingly, we detected colocalization of DR6 and caspase 6 (as well as hyperphosphorylated Tau and APP) in plaque-associated dystrophic neurites. These observations support the possible involvement of the N-APP/DR6/caspase 6 pathway in the development and progression of AD-associated pathology in DS patients. Accordingly, a similar aberrant pattern of DR6 expression was detected in sporadic AD cases with severe, endstage pathology. The presence of abnormal DR6 expression within the white matter suggests that there is also a role of this pathway in the development of white matter abnormality reported in DS brain [38,39]. In particular, a recent study [39] suggests that myelination is impaired in DS hippocampus. Interestingly, a role for DR6 signaling in oligodendrocyte maturation and myelination has recently been suggested [5,40,41]. Thus, although we did not observed detectable difference in the expression pattern of DR6 in DS patients without AD pathology, the role of DR6 in the delayed myelination in DS hippocampus requires further evaluation.

Immunocytochemical studies of postmortem fetal tissue represent one of the few available approaches for studying protein expression during human brain development, providing information about their temporal and spatial distribution that can be used to interpret functional experimental data. However, one limitation in these studies is the availability of brain tissue. An ideal experimental design (including postnatal ages ranging between 10 and 40 years of age, prior to the establishment of widespread AD neurodegeneration) is difficult to achieve, and frozen representative material is not available at all developmental ages. Another important aspect that should be taken into consideration when analyzing DS brain is that individuals with DS develop dementia with clinical and neuropathologic features similar, but not identical, to those of adults with AD without DS (that is including complex developmental abnormalities) [42-45]. Moreover, it has been suggested that oxidative stress and inflammation present early events in DS brain pathology [35].

An important issue that requires clarification is represented by the still discussed mechanism of formation of N-APP fragment and its physiological role in human brain. Guo and co-authors [14] argue in their study that APPβ and total APP are both highly stable. Other recent studies report that APP can be processed by the metalloprotease meprin β, resulting in specific N-APP fragments, which, however, did not exert significant cytotoxicity [13,46]. Moreover, recently, a novel amyloid precursor protein-processing pathway that generates secreted N-terminal fragments has been described [47].

Our study cannot resolve these complex issues; however, it provides evidence of strong DR6 expression in human hippocampus with increased IR in dystrophic neurites in DS brains in parallel with the evolution of AD-associated lesions. The detection of increased DR6 expression before endstage AD pathology and dementia are established suggests that this aberrantly increased expression of DR6 could represent an early marker of AD neuronal degeneration. Thus, premature activation of DR6 may contribute to accelerate the formation of dystrophic neurites and more extensive AD pathology through apoptosis mechanisms. However, axonal degeneration might occur via multiple pathways, which can converge on a common downstream target. Recently, a nicotinamide mononucleotide adenylyltransferase 1-sensitive pathway has been described [12]. Moreover, a novel apoptotic pathway that mediates the DR6 apoptotic signal to mitochondrial dysfunction has been reported. [9].

## Conclusions

Our findings demonstrate a developmental regulation of DR6 in human hippocampus and highlight the potential role of DR6 in specific types of disease-associated neuronal degeneration. Thus, future investigation targeting DR6 in experimental models might be worthwhile to further develop our current understanding of the role of DR6 signaling pathways in the initiation or progression of hippocampal AD-associated pathology.

## Abbreviations

AD: Alzheimer’s disease; APP: amyloid precursor protein; DR: death receptor; DR6: death receptor 6; DS: Down syndrome; GW: gestational weeks; IR: immunoreactivity; N-APP: N-terminal β-amyloid precursor protein; NeuN: neuronal nuclear; PBS: phosphate buffered saline; SDS-PAGE: sodium dodecylsulfate-polyacrylamide gel electrophoresis; SEM: standard error of the mean; SLM: stratum lacunosum-moleculare; TBST: Tris-buffered saline with TWEEN.

## Competing interests

The authors declare that they have no competing interests.

## Authors’ contributions

Immunohistochemistry, Western blotting, and data analysis were performed by JvS, AI, and JA. JvS, AI, and GGK helped EA in drafting and preparing the manuscript for submission. The overall experimental design was conceived and supervised by EA. IM and GGK helped in the selection and collection of brain tissues. All authors read and approved the final manuscript.

## Supplementary Material

Additional file 1: Figure S2 β amyloid precursor protein (APP) immunoreactivity (IR) at different ages in Down syndrome (DS) hippocampus. **A**-**B**: DS hippocampus (35 GW and 8 months) showing focal accumulation in CA1 neurons at 35 GW (insert in A) and 8 months (insert in B). **C**: adult DS hippocampus with prominent increase of APP IR throughout the hippocampus; insert in **C** shows accumulation in dystrophic neurites. Hematoxylin counterstain shows blue nuclei. Scale bar (shown in C): A-C, 400 μm. Click here for file

Additional file 2: Figure S1 DR6 immunoreactivity (IR) postnatally (2 weeks, 5 months). **A**, **C**: hippocampus (2 weeks, A and 5 months, C), showing strong DR6 IR throughout the different hippocampal subfields. **B**, **D**: neocortex (2 weeks, B and 5 months, D), showing diffuse and strong DR6 IR throughout the cortex. Hematoxylin counterstain shows blue nuclei. Stratum lacunosum-moleculare, SLM; Molecular layer–dentate gyrus, ML; dentate gyrus, DG. Scale bar (shown in D): A-C, 400 μm; D, 80 μm. Click here for file

Additional file 3: Figure S3 DR6 immunoreactivity (IR) in Alzheimer’s disease (AD). **A**-**D**: AD (stages VI) showing DR6 IR throughout the different hippocampal subfields (A) and strong DR6 expression in dystrophic neurites in CA1 (B, D arrows and insert in B) and white matter (C). **E**-**F**: colocalization (purple; arrows) of amyloid precursor protein (APP; blue) and DR6 (red) in dystrophic neuritis. **G**-**H**: colocalization (purple; arrows) of hyperphosphorylated Tau (blue) and DR6 (red) in dystrophic neurites (purple; arrows), but not in neurons containing neurofibrillary tangles (arrowhead and insert in H). Hematoxylin counterstain shows blue nuclei (A-D). Scale bar (shown in H): A, 400 μm; B, 160 μm; D, E, 40 μm; C, F-H, 30 μm. Click here for file

Additional file 4: Figure S4 Spectral analysis of double labeling of DR6 with hyperphosphorylated Tau or with β amyloid precursor protein (APP) in Alzheimer’s disease (AD) and Down syndrome (DS) adult hippocampus. **A**-**C**: double labeling of DR6 with APP in AD. A: light microscopical image of the original immunostained section showing DR6 in red and APP in blue. B and C: spectral analysis of the same section showing double stained structures in purple (arrows in B) and in yellow (arrows in C). **D-F**: double labeling of DR6 with Tau in AD. D: light microscopical image of the original immunostained section showing DR6 in red and Tau in blue. E and F: spectral analysis of the same section showing double stained structures in purple (arrows in E) and in yellow (arrows in F). **G**-**H**: double labeling of DR6 with APP in DS. G: light microscopical image of the original immunostained section showing DR6 in red and APP in blue. H: spectral analysis of the same section showing double stained structures in yellow (arrows). **I**-**J**: double labeling of DR6 with Tau in DS. I: light microscopical image of the original immunostained section showing DR6 in red and Tau in blue. J: spectral analysis of the same section showing double stained structures in yellow (arrows). Scale bar (shown in J): A-F, 80 μm; G-J, 40 μm. Click here for file
