# Supplementary figures and images for: Developmental patterns of DR6 in normal human hippocampus and in Down syndrome
Source: J Neurodev Disord. 2013 Apr 24;5(1):10. doi: 10.1186/1866-1955-5-10 (PMC3666921; doi:10.1186/1866-1955-5-10)

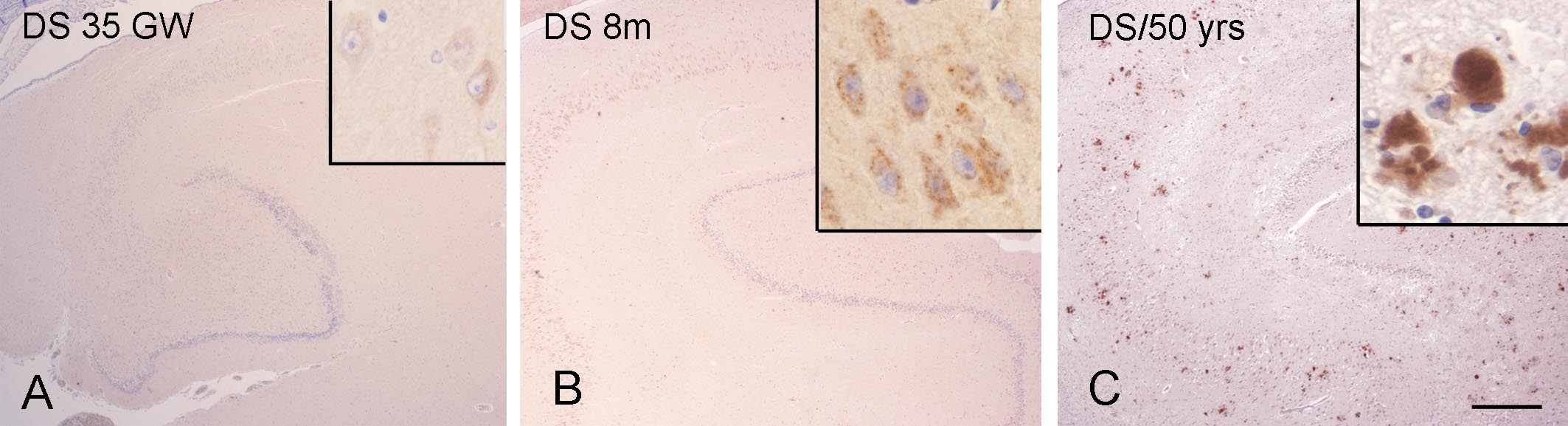

Supplement: Additional file 1: Figure S2 — β amyloid precursor protein (APP) immunoreactivity (IR) at different ages in Down syndrome (DS) hippocampus. A-B: DS hippocampus (35 GW and 8 months) showing focal accumulation in CA1 neurons at 35 GW (insert in A) and 8 months (insert in B). C: adult DS hippocampus with prominent increase of APP IR throughout the hippocampus; insert in C shows accumulation in dystrophic neurites. Hematoxylin counterstain shows blue nuclei. Scale bar (shown in C): A-C, 400 μm. [file 1866-1955-5-10-S1.jpeg]

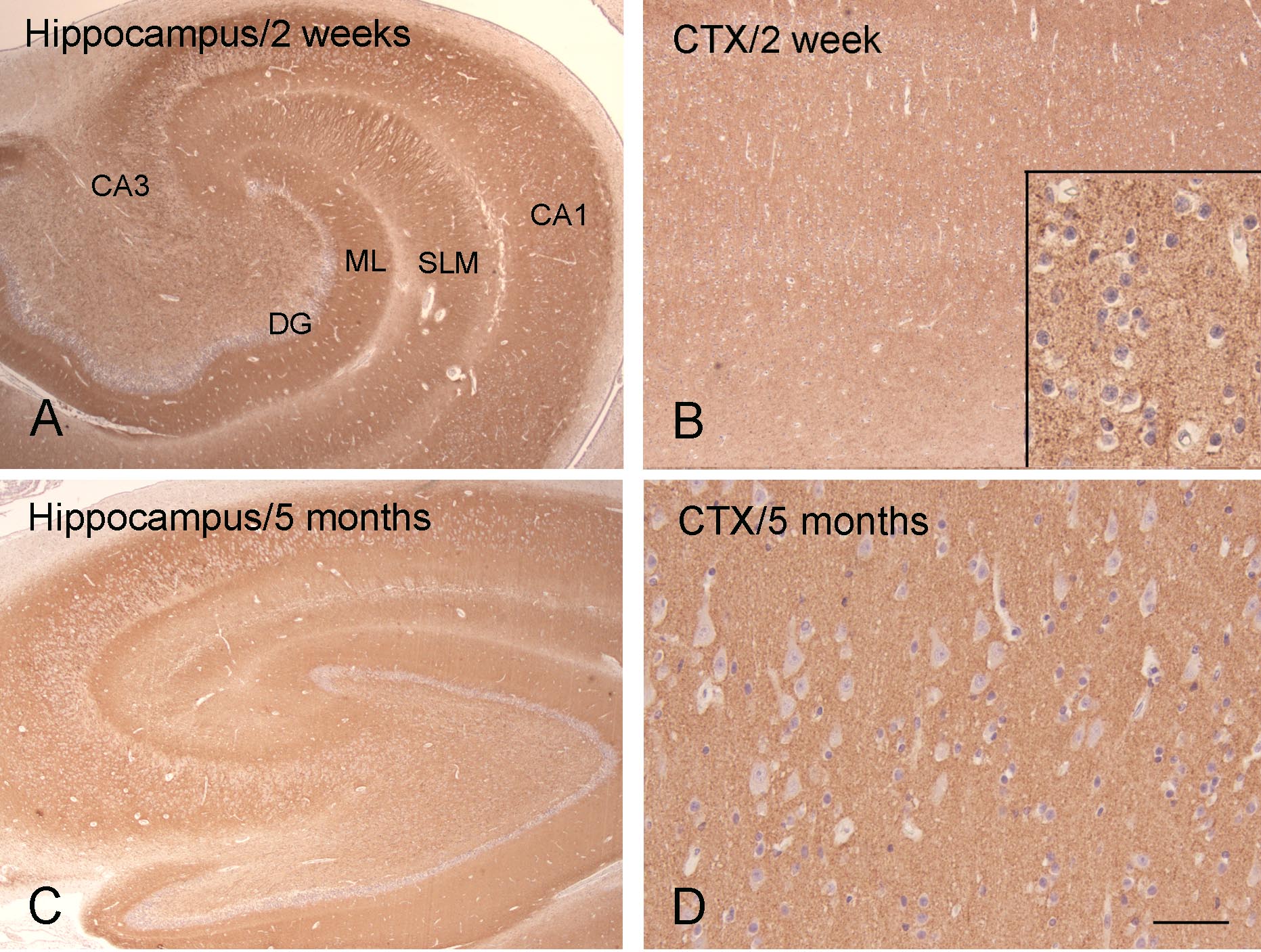

Supplement: Additional file 2: Figure S1 — DR6 immunoreactivity (IR) postnatally (2 weeks, 5 months). A, C: hippocampus (2 weeks, A and 5 months, C), showing strong DR6 IR throughout the different hippocampal subfields. B, D: neocortex (2 weeks, B and 5 months, D), showing diffuse and strong DR6 IR throughout the cortex. Hematoxylin counterstain shows blue nuclei. Stratum lacunosum-moleculare, SLM; Molecular layer–dentate gyrus, ML; dentate gyrus, DG. Scale bar (shown in D): A-C, 400 μm; D, 80 μm. [file 1866-1955-5-10-S2.jpeg]

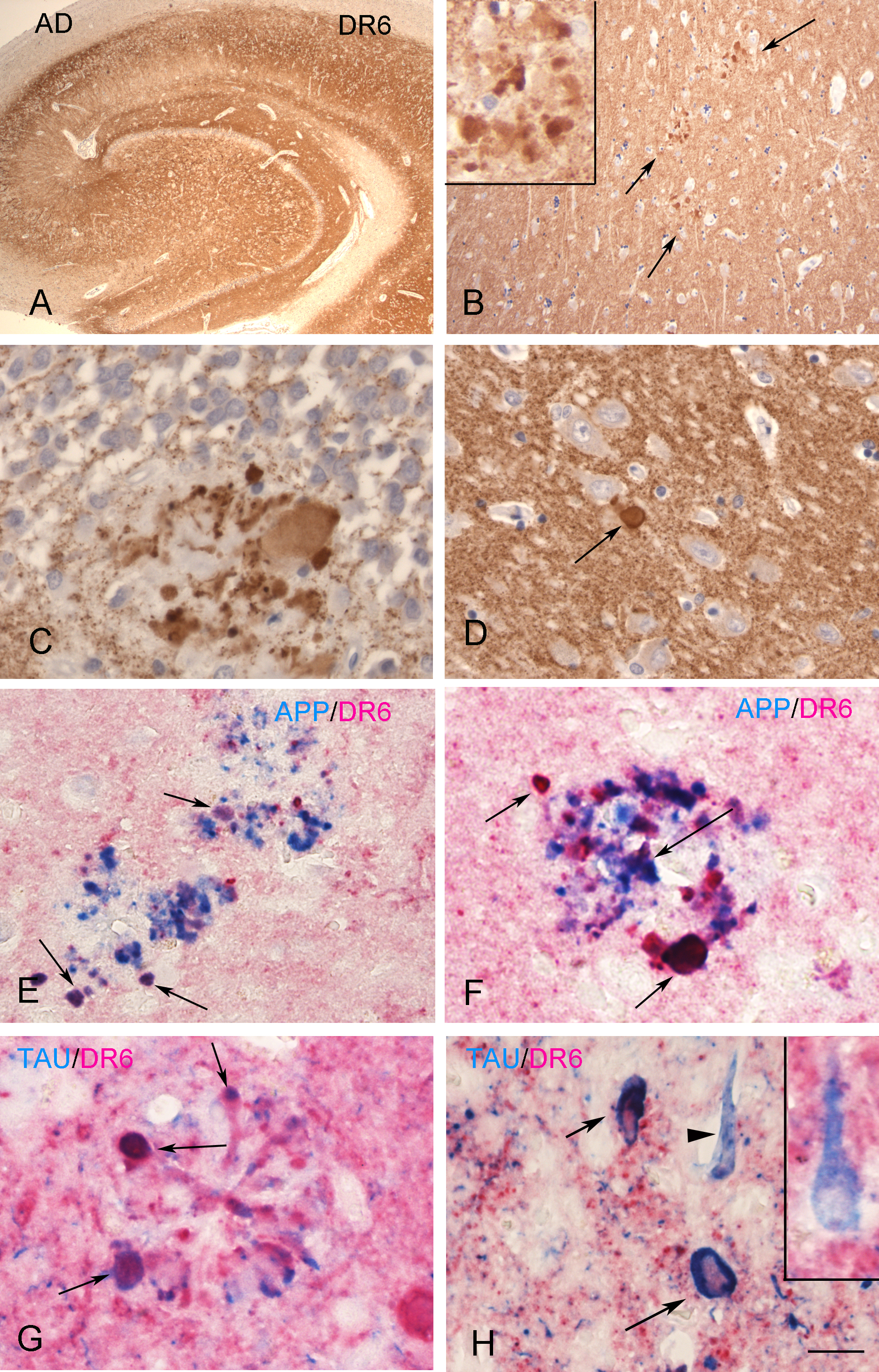

Supplement: Additional file 3: Figure S3 — DR6 immunoreactivity (IR) in Alzheimer’s disease (AD). A-D: AD (stages VI) showing DR6 IR throughout the different hippocampal subfields (A) and strong DR6 expression in dystrophic neurites in CA1 (B, D arrows and insert in B) and white matter (C). E-F: colocalization (purple; arrows) of amyloid precursor protein (APP; blue) and DR6 (red) in dystrophic neuritis. G-H: colocalization (purple; arrows) of hyperphosphorylated Tau (blue) and DR6 (red) in dystrophic neurites (purple; arrows), but not in neurons containing neurofibrillary tangles (arrowhead and insert in H). Hematoxylin counterstain shows blue nuclei (A-D). Scale bar (shown in H): A, 400 μm; B, 160 μm; D, E, 40 μm; C, F-H, 30 μm. [file 1866-1955-5-10-S3.jpeg]

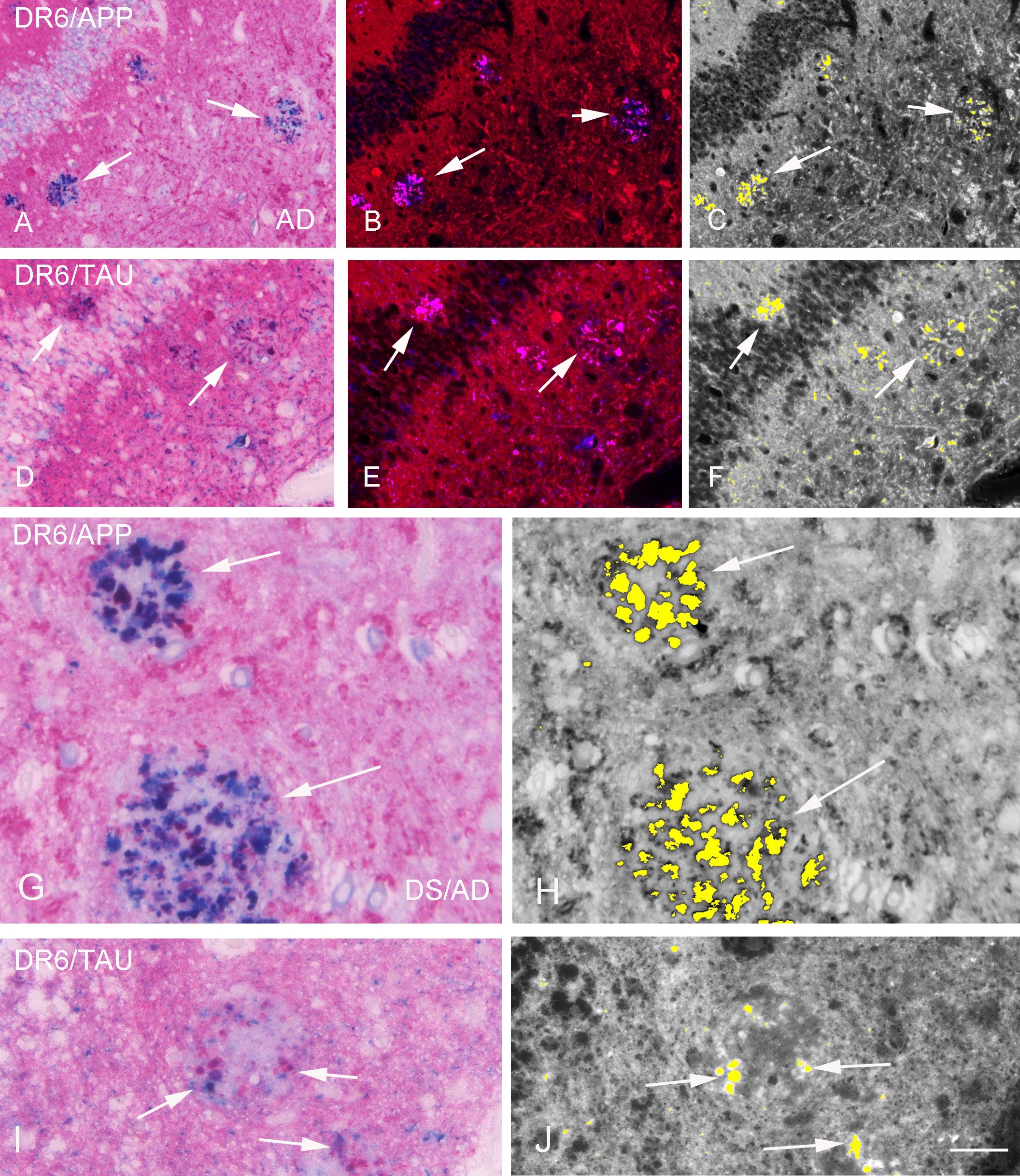

Supplement: Additional file 4: Figure S4 — Spectral analysis of double labeling of DR6 with hyperphosphorylated Tau or with β amyloid precursor protein (APP) in Alzheimer’s disease (AD) and Down syndrome (DS) adult hippocampus. A-C: double labeling of DR6 with APP in AD. A: light microscopical image of the original immunostained section showing DR6 in red and APP in blue. B and C: spectral analysis of the same section showing double stained structures in purple (arrows in B) and in yellow (arrows in C). D-F: double labeling of DR6 with Tau in AD. D: light microscopical image of the original immunostained section showing DR6 in red and Tau in blue. E and F: spectral analysis of the same section showing double stained structures in purple (arrows in E) and in yellow (arrows in F). G-H: double labeling of DR6 with APP in DS. G: light microscopical image of the original immunostained section showing DR6 in red and APP in blue. H: spectral analysis of the same section showing double stained structures in yellow (arrows). I-J: double labeling of DR6 with Tau in DS. I: light microscopical image of the original immunostained section showing DR6 in red and Tau in blue. J: spectral analysis of the same section showing double stained structures in yellow (arrows). Scale bar (shown in J): A-F, 80 μm; G-J, 40 μm. [file 1866-1955-5-10-S4.jpeg]
